# Contribution of different frass fertilizer products on enhanced growth, yield and nutrient quality of Broccoli [*Brassica oleracea*]

**DOI:** 10.3389/fpls.2025.1613814

**Published:** 2025-08-14

**Authors:** Noella Kagehi, Dennis Beesigamukama, Chrysantus M. Tanga, Mathew P. Ngugi, Sevgan Subramanian, Fathiya M. Khamis

**Affiliations:** ^1^ International Center of Insect Physiology and Ecology, Nairobi, Kenya; ^2^ Department of Microbiology, Biochemistry, and Biotechnology, Kenyatta University, Nairobi, Kenya

**Keywords:** nutrient recycling, insect frass fertilizer, soil health, broccoli yield, regenerative agriculture, economic returns

## Abstract

Vegetables are crucial for food security and income, but in developing countries their production is hindered by low soil fertility. Although the insect frass fertilizer is a potential solution, its use is constrained by limited product choices. Unlike conventional fertilizers, which are available in different forms, the insect frass fertilizer is mostly available in solid form. Here, we evaluated the effects of different black soldier fly frass fertilizer (BSFFF) products on broccoli [*Brassica oleracea*] growth, yield, and nutritional profiles. Solid, liquid BSFFF, chitin-fortified solid BSFFF, chitin-fortified liquid BSFFF, and commercial organic fertilizer (Safi) were applied at rates equivalent to 250 kg N ha^-1^ for two cropping seasons. The control treatment consisted of unfertilized soil. Results showed that solid and chitin-fortified solid BSFFF products significantly increased broccoli leaf growth and chlorophyll concentration by 54% and 11%, respectively, compared to the other BSFFF products. Soils amended with BSFFF products produced broccoli with higher number of heads (28 – 158%), fresh yield (26 – 138%), dry yield (17 – 60%), and aboveground biomass (7 – 117%) compared to Safi and control treatments. Broccoli grown in soil amended with BSFFF had higher nitrogen (84%), phosphorus (93%), potassium (51%) uptake, and agronomic use efficiency (4.6-fold) as compared to Safi and the control. Additionally, the application liquid BSFFF produced broccoli heads with higher levels of crude fat (61%), carbohydrates (16%), and calcium (38%) compared to other BSFFF products. Conversely, broccoli grown using chitin-fortified BSFFF exhibited the highest levels of crude protein, potassium, crush ash, and phosphorus. The net income and gross margin achieved with BSFFF treatments were 19 – 26-fold and 29 – 63-fold higher than values obtained Safi, respectively, with higher profitability achieved using chitin-fortified BSFFF formulations. These findings demonstrate the efficacy of different BSFFF formulations in supporting circular economy for safe vegetable production, and improved food and nutrition security.

## 1 Introduction

Broccoli is an edible green plant belonging to the cabbage family and is highly nutritious, offering numerous health advantages due to its rich content of nutrients. It provides essential nutrients such as magnesium, calcium, iron, potassium, vitamins C, D, and K, folic acid, and dietary fiber (Michaud et al., 2002). In comparison to other vegetables, broccoli supports immune health, promotes bone and skin health, helps reduce cholesterol levels, and plays a critical role in cancer prevention (Mohamed et al., 2021). China and India together account for approximately 73% of the world’s total broccoli production, while Kenya is one of the leading exporters in Africa (Omondi, 2014). The optimal production of broccoli requires fertile soils with sufficient supply of nitrogen, excess and insufficient nitrogen application may cause yield reduction, physiological disorders, and pathological problems (Yildirim et al., 2020).

Sub-Saharan Africa’s (SSA) crop production faces significant obstacles due to inadequate soil fertility, compounded by the limited use of mineral fertilizers, which are often expensive and scarce in local markets (FAO et al., 2024). Therefore, the low broccoli yields in some SSA countries like Kenya, Uganda, and Tanzania can be largely attributed to the low soil fertility levels (Raimi et al., 2017; Tully et al., 2015). Previous studies have shown that organic fertilizers tend to give better results in broccoli cultivation in terms of broccoli yield, growth parameters, and nutrient uptake (Altuntaş, 2018). This is because organic fertilizers supply the crucial macronutrients required for broccoli development, uptake and utilization of macronutrients, and boosts soil microbial activity (Islam et al., 2004; Wortmann et al., 2019). The integrated use of organic and inorganic fertilizers enhances and preserves soil fertility, boosts crop returns, and maximizes nutrient utilization efficacy in Sub-Saharan Africa (Vanlauwe et al., 2015). Despite these benefits, many farmers in SSA don’t use organic fertilizers due to competitive uses (Rufino et al., 2011).

Insect farming is emerging as a sustainable method to accelerate the recycling of low-value organic waste streams into high-value products, such as organic fertilizer within 5 weeks (Beesigamukama et al., 2021a, 2023; Beesigamukama et al., 2022a). The black soldier fly (BSF) frass fertilizer is an organic product consisting of a mixture of insect excretion, exoskeleton, and substrate residue leftovers at the end of larval growth with demonstrated potential for soil health management and crop production (Beesigamukama et al., 2023; Poveda, 2021). As opposed to ordinary composts, the insect frass fertilizer is richer in beneficial microbes, and plant nutrients (Terfa, 2021), free of phytotoxicity, and contributes to pests and pathogens suppression (Beesigamukama et al., 2023).

The BSF contains chitin content (Lagat et al., 2021) that has been found to suppress pathogens and soil-borne pests such as nematodes (Anedo et al., 2025; Kisaakye et al., 2024). Chitin also enhances the abundance of plant growth-promoting fungi and rhizal bacteria, which play crucial roles in nitrogen fixation, phosphate solubilization, phytochrome production, photosynthesis, and plant protection against abiotic stress (van de Zande et al., 2024; Shahrajabian et al., 2021; Shamshina et al., 2020). As a result, the application of BSF frass fertilizer (BSFFF) boosts the growth and yield of different crops, including vegetables, cereals, tubers, and pastures (LoMonaco et al., 2024; Abiya et al., 2022; Dzepe et al., 2022; Anyega et al., 2021; Menino et al., 2021; Beesigamukama et al., 2020a; Quilliam et al., 2020).

Unlike the mineral and conventional organic fertilizers, which are available in both solid and liquid forms, research on BSF frass fertilizer has largely focused on the solid form, limiting its uptake and application in addressing different production challenges. To address this gap, the International Centre of Insect Physiology and Ecology (*icipe*) and its partners have developed diversified frass fertilizer products, including powered, granulated, liquid, and chitin-fortified BSF frass fertilizers in response to consumer demands, economic conditions, and biotic and abiotic challenges to crop production (Tanga and Kababu, 2023). The efficacy of chitin-fortified frass fertilizers has been demonstrated against soil-borne pests (Anedo et al., 2025; Kisaakye et al., 2024).However, the comparative performance of different BSF frass fertilizer products and formulations (liquid, solid, and chitin-fortified) has not been assessed, yet such information is crucial in selecting the suitable products for supporting specific crops and in different production environments (Beesigamukama et al., 2023). Moreover, the performance of the different forms of BSFFF for broccoli growth, yield, and nutritional profiles in comparison to existing organic fertilizers is still unknown. The present study aimed to evaluate the impact of different BSFFF products on the growth, yield, and nutritional value of broccoli, in comparison with commercial organic fertilizers, to generate recommendations for integrating different forms of BSFFF into existing farming practices for improved soil health and crop productivity.

## 2 Materials and methods

### 2.1 Description of the experimental site

The Field experiments were conducted at the International Centre of Insect Physiology and Ecology (*icipe*), situated in Nairobi (1° 13’ 18.5” S 36° 53’ 50.7’’ E; 1616 m above sea level) over two cropping seasons (June – October 2023 and November – March 2024). The site experiences an average monthly temperature range of 12 – 29°C and receives an average annual rainfall of 787 mm characterized by a bimodal distribution. The short rains typically occur from October to December, while the long rains span from March to June. The study area has well-drained, mostly sandy clay soils, classified as humic Nitisols. There is 2400 mm of evapotranspiration each year (Jaetzold and Schmidt, 1982).

### 2.2 Soil sampling and land preparation

From each plot, the soil was collected from 0–30 cm depth before the application of fertilizers using a soil auger and following the zig-zag pattern. The representative soil samples were collected by quarter sampling. The collected soil was air-dried for 5 days at 25°C, and ground using a mortar and pestle before sieving through a 2 mm sieve to remove foreign objects and bigger particles. The soil was analyzed for physiochemical properties following standard methods described in Okalebo et al. (2002). The results of soil analysis are presented in **Table 1**. The land was prepared by hand hoeing to remove all the weeds and create a fine tilth for broccoli cultivation.

**Table 1:** Physical-chemical characteristics of the test soil and organic fertilizers used in the study.

Parameter	Soil	Solid BSFFF	Liquid BSFFF	Chitin-fortified solid BSFFF	Chitin-fortified liquid BSFFF	Safi
pH	5.9	7.7	5.9	7.0	5.8	6.4
Electrical conductivity (mS/cm)	0.105	11.8	38.1	4.39	32.9	6.1
Organic carbon (%)	0.9	43.9	1.09	42.0	1.3	45.1
Nitrogen (%)	0.12	3.69	0.10	4.49	0.14	3.0
Phosphorus (%)	0.001	1.54	0.11	0.61	0.07	1.23
Potassium (%)	0.08	2.37	0.79	2.2	0.48	1.49
Calcium (%)	0.22	1.00	0.03	4.99	0.05	0.29
Magnesium (%)	0.048	0.59	0.05	0.39	0.04	0.43
Sulphur (mg/kg)	17.6	4350	294	3233	156.7	–
Manganese (mg/kg)	452.3	203	16.1	1403.3	23.4	–
Iron (mg/kg)	104.3	4985	55.1	9610	31.9	–
Copper (mg/kg)	1.59	15.5	0.18	15.6	0.01	–
Zinc (mg/kg)	13.0	61.5	0.73	144.7	0.01	–
Boron (mg/kg)	0.90	42.9	0.84	28.1	0.85	–
C/N ratio	7.5	11.9	10.8	7.61	8.6	15
Ammonium (mg/kg)	47.6	–	8643	–	1720	39.4
Nitrate (mg/kg)	18.9	–	602.7	–	458.7	92.3
Cation exchange capacity (Cmol/kg)	19.7	–		–		–
Textural class	loam	–		–		–

### 2.3 Source of materials

The trial comprised of five fertilizers: solid BSFFF, solid BSFFF fortified with 3% BSF chitin, liquid BSFFF, and liquid BSFFF fortified with 3% BSF chitin, and commercial organic fertilizer (Safi). The liquid and solid BSFFF used in the field experiment were prepared at *icipe* following procedures described by Anedo et al. (2025) and Kisaakye et al. (2024). The solid BSF frass fertilizer was a product obtained from the feeding of BSF larvae on a substrate made of potato peels at *icipe*, Nairobi, Kenya. The BSF larvae were reared according to Shumo et al. (2019). After harvesting of larvae at 2 weeks, the frass was composted for 4 weeks using the heap method to obtain a mature and stable frass product, which was used in this study as solid BSF frass fertilizer. The chitin-fortified liquid fertilizer was formulated using black soldier fly frass, BSF exuviae effective microorganisms (EMs), biochar and molasses. The mixed solid materials were placed in airtight fermentation tanks as described by Kisaakye et al. (2024). Each tank was tightly covered with a lid fitted with a 5-mm-diameter tube to allow for gaseous exchange. The contents were fermented for 6 weeks with weekly stirring to obtain mature and stable chitin-fortified fertilizer. The maturity and stability of frass fertilizer extracts were determined following previously described procedures (Beesigamukama et al., 2021a). After fermentation, the chitin-fortified fertilizer was sieved through a 150-µm sieve (Endecotts Ltd., London, UK), put into 20-L non-transparent jerrycans, and stored in a cool place pending field experiment. The liquid BSF frass fertilizer was prepared using the same procedure, excluding addition of chitin. The chitin-fortified solid fertilizer was produced by mixing the composted BSF frass fertilizer with milled BSF exuviae following procedures described by Anedo et al. (2025). The commercial organic fertilizer (Safi) was obtained from Safi Organics Limited located in Mwea town, Kirinyaga county, Kenya. The organic fertilizers were analyzed for nutrient levels and other properties following standard laboratory methods described in Okalebo et al. (2002), and the results presented in **Table 1**. Broccoli seeds (Green sprouting variety) [*Brassica oleracea*] were obtained from SimLaw seeds, Nairobi, Kenya. The seeds were sown in 4 seedling trays of 160 wells. The trays were filled with topsoil obtained from *icipe* farm and then planted with one seed per well. The trays were placed in a greenhouse watered twice a day and managed following recommended practices (Omondi, 2014). The seedlings were then transplanted into the experimental plots 4 weeks after germination.

### 2.4 Experimental design and treatments

A randomized complete block design (RCBD) with three replications was used to establish the experiment. The RCBD was chosen to cater for soil heterogeneity. Plots of 2 m × 3 m with a spacing of 1 m between blocks and 0.5 m between plots were used. The solid organic fertilizers were applied 3 days before planting, using band placement method to ensure the timely onset of mineralization and synchrony of nutrient release for plant uptake. The liquid BSFFF and chitin-fortified liquid BSFFF were applied twice weekly, starting from the 3^rd^ week after transplanting up to the harvesting time. This was achieved by diluting 200 ml of each liquid fertilizer into 20 liters of water. The split-application was meant to reduce nutrient leaching and improve fertilizer use efficiency. The liquid fertilizers were applied by drenching the soil around the plant base using a knapsack sprayer. The fertilizers were applied at rates equivalent to 250 kg N/ha (Vågen et al., 2007). To meet this nitrogen requirement, the amounts applied were6775.1 kg/ha, 5567.9 kg/ha, and 8333.3 kg/ha for solid BSFFF, solid BSFFF fortified with 3% BSF chitin, and Safi, respectively. Broccoli was planted at a spacing of 60 cm between rows by 45 cm between plants.

During the experiment, weeding was done twice a month using a hand hoe, while irrigation was performed three times per week. The study was conducted in the same plots for two consecutive cropping seasons. The first season ran from June to October 2023 while the second season began in December 2023 and ended in April 2024. For clarity, throughout this manuscript, the seasons are referred to as season 2023A and season 2023B for the first and second seasons, respectively.

### 2.5 Broccoli growth parameters

Broccoli growth was assessed by collecting data on plant height, chlorophyll content, stem diameter, leaf length, leaf width, number of secondary curbs/heads, and the number of leaves every week, from the 8^th^ week after transplanting up to the 17^th^ week when harvesting was conducted. Ten randomly selected plants were tagged per plot and used to measure the different growth parameters. The plant height, leaf length, and width were measured using a tape measure. Plant height was measured using a tape measure that was stretched from the ground level up to the tip of the shoot. Leaf chlorophyll was measured using SPAD-502 (Konica Minolta, Tokyo, Japan), which was placed on top of six fully grown leaves. Digital vernier callipers (Mitutoyo, Kanagawa, Japan) were used to measure the stem diameter at 10 cm from the ground.

The total biomass and yield were determined at the harvesting stage. At harvesting, the plants from each plot were uprooted and soil washed off from the roots using tap water. The edible flower heads were harvested by cutting using a knife and non-edible parts were discarded. The yield was determined by determining the weight of heads per treatment using a weighing balance. Part of the harvested broccoli heads was chopped, air-dried to reduce moisture for 5 days, and then oven-dried (SDO-225-CLAD-F-200-HYD, Wagtech Projects Limited, Thatcham, United Kingdom) at 60°C for 72 hours to drive off all the moisture and determine dry matter yield on a kg per hectare basis. The dried samples were then crushed into powder using an analytical mill to measure proximate (crude ash, carbohydrates, crude fiber, crude fat, and crude proteins using standard methods (Van Soest et al., 1991). The minerals were determined using atomic absorption spectrometry (AAS) (iCE 3300 AA system, Thermo Scientific, Beijing, China) at respective wavelengths (Okalebo et al., 2002).

### 2.6 Nutrient uptake and use efficiency of broccoli

The concentrations of N, P, and K were determined in section 2.5 (Okalebo et al., 2002), and yield data were used to calculate nutrient uptake in broccoli heads (Equation 1). Agronomic efficiency (AEN), which is a measure of yield produced per unit N supplied from each treatment, was also calculated using broccoli yield from each treatment (Equation 2).


(1) $$\begin{array}{c} Nutrient\;\left(N,\;P\;and\;K\right)\;uptake\;\left(kg\;ha^{-1}\right)\\ =\frac{Nutrient\;concentration\;\left(\%\right)\;\times \;broccoli\;head\;dry\;yield\;\left(kg\;ha^{-1}\right)}{100}\; \end{array}$$

(2) $$\begin{array}{c} AEN\left(kg\;kg\;N^{-1}\right)\\ =\frac{\left[Dry\;yield_{F}\;\left(kg\;ha^{-1}\right)\;-\;Dry\;yield_{C}\;\left(kg\;ha^{-1}\right)\right]}{Quality\;of\;N\;applied\;\left(kg\;ha^{-1}\right)} \end{array}$$

Where,

F represents plots that received fertilizer treatments

C represents the control treatment (unfertilized soil)

### 2.7 Economic returns to broccoli production

The profitability of broccoli production using the different forms of BSFFF and commercial organic fertilizer was assessed by determining net income, benefit-to-cost ratio, return on investment, and gross margins (Equations 3-6) (Chia et al., 2019). The variable costs considered during the study included the cost of seeds, fertilizers, labor, and pesticides. The pesticide was used to manage aphids during the experiment. We used Degree Max 200EC (Osho Chemical Industries limited, Nairobi, Kenya). The application rates were 2.5ml per 20 liters of water, and it was applied once a week for 5 weeks. The prices of solid fertilizers were sourced from the websites of Mzuri Organics (https://mzuriorganics.co.ke) and Safi Organics (https://safiorganics.co.ke) while the price of green sprouting broccoli seeds was obtained from Simlaw seeds (https://www.simlaw.co.ke). The labor costs during land preparation, fertilizer application, planting, and weeding were determined using the hourly rate of US$1.25 (The Labor Institutions Act (No. 12 of 2007), 2022). Broccoli fresh yield was considered a source of revenue.


(3) Net income=Gross income–Total cost of production

(4) $$Benefit\;cost\;ratio=\frac{Net\;income}{Total\;cost\;of\;production}\;$$

(5) $$Gross\;margin\;\left(\%\right)=\frac{Net\;income}{Gross\;income}*100$$

(6) $$Return\;on\;investment\;\left(\%\right)=\frac{Net\;income}{Total\;cost\;of\;production}*100$$

### 2.8 Statistical analysis

The Shapiro-Wilk test was used to check the normality of data. Data on broccoli growth (number of leaves, leaf area, plant height, number of curds, chlorophyll content, and stem diameter) parameters were analyzed using the linear mixed effect function from the model with “lmer” package “lme4” in R Studio (R Core Team, 2022) with fertilizer treatment and growth period as the fixed effect, replication as a random effect). The following data were analyzed using Analysis of Variance (ANOVA) because they met the assumptions of normality: broccoli growth parameters, including plant height, number of leaves, leaf area, stem diameter, and chlorophyll concentration for both seasons. Also, the number of edible flower heads, fresh and dry yield, proximate composition of broccoli (crude protein, ash, fat, fiber, and carbohydrates), mineral content (potassium, magnesium, calcium, sodium, manganese, copper, boron, molybdenum, zinc, sulfur, and cobalt), nutrient uptake (potassium and phosphorus for both seasons), and agronomic nitrogen use efficiency for season 2023B, were analyzed using (ANOVA). Conversely, data that did not meet normality assumptions [fresh biomass (2023A), dry biomass (2023A and 2023B), nitrogen uptake and agronomic nitrogen use efficiency (2023A), phosphorus and iron concentrations in broccoli, economic returns (2023B), benefit-to-cost ratio and return on investment (2023A), and dry matter content] were analyzed using a Generalized Linear Model (GLM). In case of significant differences, the means were separated using Tukey’s Honest Significant Difference (HSD) test. All statistical analyses were conducted separately for each season using R software version 3.6.1 for Windows (R Core Team, 2022).

## 3 Results

### 3.1 Impact of BSF frass fertilizers and commercial fertilizer on broccoli growth

#### 3.1.1 Plant height and leaf growth

The fertilizer treatments caused significant differences in broccoli height (season 2023A: χ2 = 44.52, df = 5, p<0.0 01, season 2023B: χ2 = 95.4, df = 5, p< 0.001). The influence of growth period was significant only during season 2023A only (season 2023A: χ2 = 151.6, df = 1, p< 0.001, season 2023B: χ2 = 1.2, df = 1, p = 0.28). The interaction between the fertilizer formulations and growth period was significant during season 2023B only (season 2023A: χ2 = 7.64, df = 5, p = 0.18, season 2023B: χ2 = 12.9, df = 5, p< 0.05).

**Figure 1:**
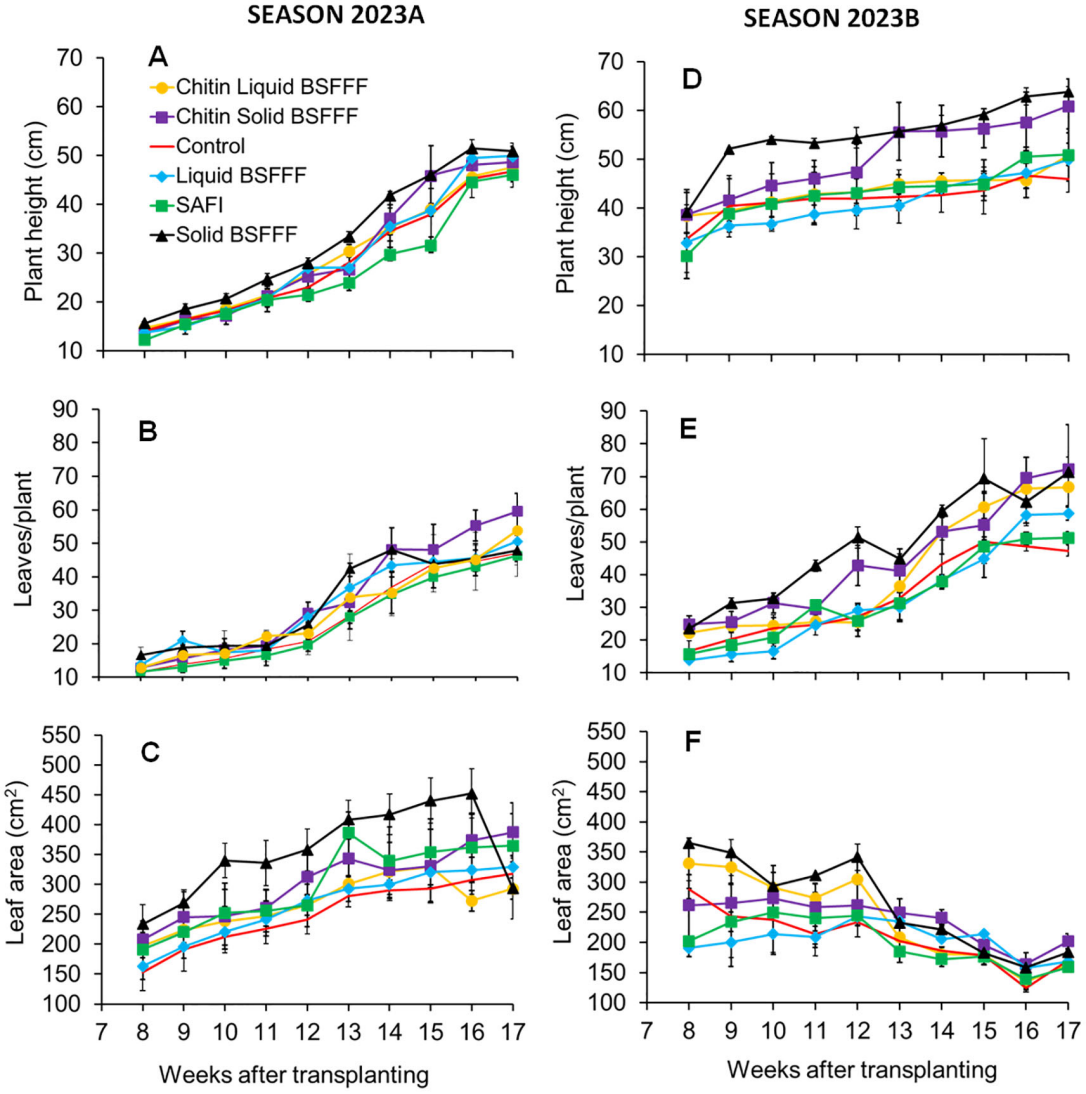
Trends in plant height **(A, D)**, number of leaves **(B, E)**, and leaf area **(C, F)**, of broccoli grown in soil amended with BSF frass fertilizers and commercial fertilizer during Season 2023A **(A-C)** and Season 2023B **(D-F)**. BSFFF, black soldier fly frass fertilizer; Safi, commercial organic fertilizer; control, no fertilizer amendment.

Plant heights followed an increasing trend up to peak values in the 16^th^week (12-50cm) (F = 1.5, df = 5, p = 0.27) and 17^th^ week (30 – 63cm) (F = 1.7, df = 5, p = 0.21) during the seasons 2023A and 2023B, respectively. However, these increases were not significant (**Figures 1A, D**). The broccoli height was 47–51 cm at the end of season 2023A (F = 0.57, df = 5, p = 0.73), with plots amended with solid BSFFF producing broccoli with the highest plant height, with control the least height. It was noted that these differences were not significant. At the end of season 2023B (F = 1.7, df = 5, p = 0.21), the plant height ranged between 46 and 64 cm. Plots amended with solid BSFFF produced the tallest plants while plants grown in the control plots were the shortest. Generally, higher values of plant heights were observed in the second season compared to the first season.

The fertilizer amendments caused significant differences in broccoli leaf number (season 2023A: χ2 = 23.99, df = 5, p< 0.001, season 2023B: χ2 = 161, df = 5, p< 0.001), and growth period (season 2023A: χ2 = 659.6, df = 1, p< 0.001, season 2023B: χ2 = 720.5, df = 1, p< 0.001). The combined effect was significant in the season 2023B only (season 2023A: χ2 = 7.7, df = 5, p = 0.17, season 2023B: χ2 = 10.5, df = 5, p< 0.1).

An increasing trend in the number of leaves was observed in both seasons, reaching peak levels in the 17^th^ week, however these increases were not statistically significant (season 2023A: F = 1.4, df = 5, p = 0.28, season 2023B: F = 2.6, df = 5, p = 0.082) (**Figures 1B, E**). At the end of season 2023A, the number of leaves ranged between 46 and 60, with broccoli grown in plots treated with chitin-fortified solid BSFFF achieving the highest count, while Safi-treated plants had the lowest. Similarly, the chitin-fortified solid BSFFF produced plants with the highest leaf number (72) at the end of season 2023B while the control-treated plants had the fewest leaves (47).

The broccoli leaf area showed significant variations due to fertilizer treatments (season 2023A: χ2 = 71.93, df = 5, p< 0.001, season 2023B: χ2 = 58.5, df = 5, p< 0.001), and the growth time (season 2023A: χ2 = 115.74, df = 1, p< 0.001, season 2023B: χ2 = 172.8, df = 1, p< 0.001). The combined effect was significant in season 2023B only (season 2023A: χ2 = 4.37, df = 5, p = 0.49, season 2023B: χ2 = 42.2, df = 5, p< 0.001).

The leaf area increased through season 2023A, but a declining trend was observed from the 12th week after transplanting up to the end of experiments during season 2023B, although this decline was not significant (season 2023B: F = 2.7, df = 5, p = 0.07). Plots treated with solid BSFFF maintained higher leaf area from the 8^th^ week (season 2023A: F = 0.9, df = 5, p = 0.47) to the 16^th^ week (season 2023A: F = 2.9, df = 5, p = 0.062) and the 8^th^ week which was significant (season 2023B: F = 6, df = 5, p< 0.01) to the 12^th^ week (season 2023B: F = 2.7, df = 5, p = 0.07) during seasons 2023A and 2023B, respectively (**Figures 1C, F**). The leaf area ranged from 292.7 to 387 cm² in season 2023A and 159 to 201 cm² in season 2023B, with chitin-fortified solid BSFFF amendment achieving the largest broccoli leaf area in both seasons. On the other hand, chitin-fortified liquid BSFFF and Safi producing leaves with the least areas at the end of both seasons, respectively.

#### 3.1.2 Stem diameter and leaf chlorophyll

The broccoli stem diameter showed significant differences owing to fertilizer amendments (season 2023A: χ2 = 16.13, df = 5, p< 0.01, season 2023B: χ2 = 67.0, df = 5, p< 0.001) and growth period (season 2023A: χ2 = 210.48, df = 1, p< 0.001, season 2023B: χ2 = 168.31, df = 1, p< 0.001).The combined effect was insignificant in both seasons (season 2023A: χ2 = 6.06, df = 5, p = 0.3, season 2023B: χ2 = 0.48, df = 5, p = 0.99).

**Figure 2:**
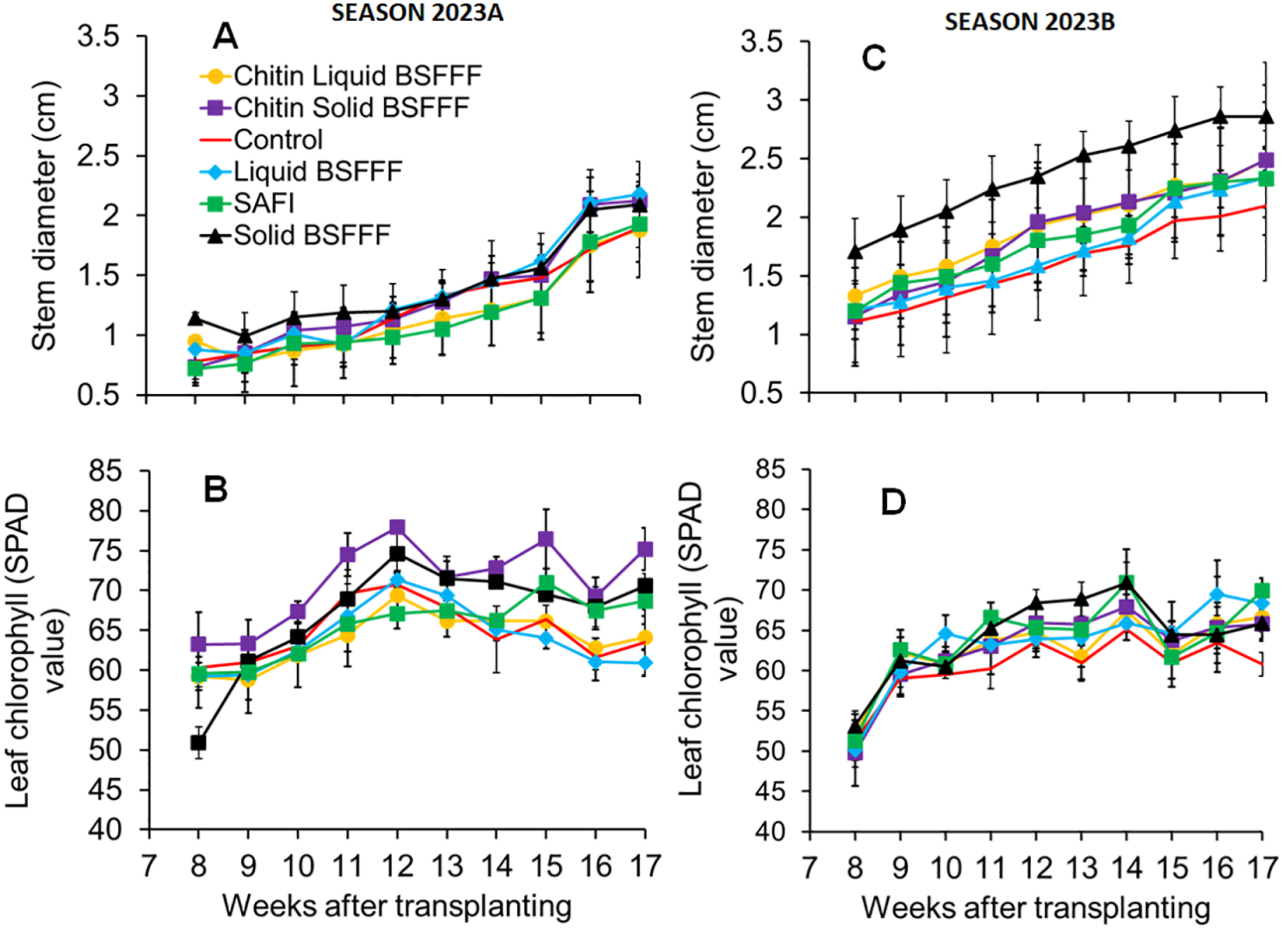
Effects of BSF Frass Fertilizers and commercial fertilizer on broccoli stem diameter **(A, C)**, and chlorophyll concentration **(B, D)** during Season 2023A **(A, B)** and Season 2023B **(C, D)**. BSFFF, black soldier fly frass fertilizer; Safi, commercial organic fertilizer; control, no fertilizer amendment.

An increasing trend was observed throughout the experiments in both growing seasons (**Figures 2A, C**). The stem diameter varied between 1.9 and 2.2 cm at the end of season 2023A, and 2.1 – 2.9 cm at the end of season 2023B. Plants grown using chitin-fortified solid BSFFF and control had the smallest stem diameter in seasons 2023A and 2023B, respectively. The largest stem diameter in season 2023A was achieved by liquid BSFFF treatment (F = 0.78, df = 5, p = 0.58) during the 17th week. Similarly, in season 2023B, the solid BSFFF treatment produced the largest stem diameter at the 17^th^ week, although this was not significant (F = 0.34, df = 5, p = 0.88).

Broccoli leaf chlorophyll concentration differed significantly due to fertilizer treatments in (season 2023A: χ2 = 54.12, df = 5, p< 0.001, season 2023B: χ2 = 4.43, df = 5, p< 0.001) and growth period (season 2023A: χ2 = 31.18, df = 1, p< 0.001, season 2023B: χ2 = 68.8, df = 1, p< 0.001). The interaction between the growth stage and fertilizer amendments was significant in season 2023A only (season 2023A: χ2 = 11.02, df = 5, p< 0.1, season 2023B: χ2 = 1.48, df = 5, p = 0.91).

An increasing thread in the leaf chlorophyll content was observed from the 8^th^ (F = 0.3, df = 5, p = 0.28) to the 12^th^ week (F = 3.2, df = 5, p< 0.05) and from the 8^th^ week (F = 0.2, df = 5, p = 0.96) to the 14^th^ week (F = 0.6, df = 5, p = 0.685) after transplanting during seasons 2023A and 2023B, respectively (**Figures 2B, D**). During season 2023A, the highest chlorophyll concentration was achieved in the 12^th^ week using chitin-fortified solid BSFFF. In season 2023B, the highest chlorophyll concentration was recorded in the 14^th^ week after transplanting from plots amended with solid BSFFF, but this was not significantly different from the values achieved using other treatments.

The BSFFF amendments significantly increased the chlorophyll concentration by 2 – 19% and 3 – 9% relative to control and Safi, respectively, at the end of season 2023A (F = 4.8, df = 5, p< 0.05). The application of solid BSFFF in season 2023A produced broccoli with 16% chlorophyll content compared with the liquid BSFFF treatment. Also, the chitin-fortified liquid BSFFF produced broccoli with 3.2-fold higher leaf chlorophyll concentration compared to liquid BSFFF. Notably, the chitin-fortified solid BSFFF enhanced leaf chlorophyll by 7% relative to solid BSFFF during season 2023A. At the end of season 2023B, the chlorophyl concentration did not vary significantly (F = 0.8, df = 5, p = 0.06), with the values ranging between 61 and 70 SPAD values for the control and Safi treatments, respectively.

### 3.2 Effect of different BSF frass fertilizer formulations and commercial fertilizer on broccoli nutrient uptake and nitrogen use efficiency

#### 3.2.1 Nitrogen uptake

The fertilizer amendments significantly influenced the nitrogen uptake of broccoli in season 2023A only (**Table 2**). The fertilizer amendments significantly increased nitrogen uptake by 18 – 84% during season 2023A and 3 – 71% during season 2023B compared to control and Safi treatments. Broccoli grown in solid BSFFF had 17% and 46% higher nitrogen uptake during season 2023A and 2023B, respectively, compared to liquid BSFFF treatments. Plots amended with chitin-fortified solid BSFFF had 33% higher nitrogen uptake than solid BSFFF in season 2023A. On the other hand, chitin-fortified liquid BSFFF produced broccoli with higher nitrogen capacity by 28% in season 2023A compared to the values achieved using liquid BSFFF. Plots with chitin-fortified solid BSFFF and solid BSFFF produced broccoli with 84% higher nitrogen uptake than other treatments during the season.

**Table 2:** Nutrient uptake and use efficiency of broccoli grown amended with BSF frass fertilizers and commercial organic fertilizer.

Fertilizer treatments	Season 2023A				Season 2023B			
	Nitrogen uptake (kg/ha)	Potassium uptake (kg/ha)	Phosphorus uptake (kg/ha)	Agronomic nitrogen efficiency (kg/kg N)	Nitrogen uptake (kg/ha)	Potassium uptake (kg/ha)	Phosphorus uptake (kg/ha)	Agronomic nitrogen efficiency (kg/kg N)
Control	12.4±2.31a	7.8±1.5a	1.4±0.41a	–	19.0±6.5a	8.7±3.05a	1.9±0.71a	0.00±1.7ab
Safi	16.8±3.1ab	9.0±0.67a	2.1±0.12a	-0.26± 0.66a	15.0±3.22ab	7.7±1.49a	1.7±0.33a	-0.48±0.05a
Solid BSFFF	17.2±1.14ab	9.5±0.64a	2.2±0.26a	1.58±0.43ab	28.4±2.44ab	13.3±1.01a	3.0±0.29a	4.1±0.45c
Liquid BSFFF	14.7±3.1ab	8.9±1.29a	1.9±0.39a	1.76±0.06ab	19.5±6.9ab	8.7±2.87a	2.0±0.62a	1.0±0.37bc
Chitin-fortified Solid BSFFF	22.8±2.88b	11.8±1.44a	2.8±o.31a	3.33±1.11b	25.7±5.61b	11.5±2.3a	2.6±0.54a	3.1±0.75bc
Chitin-fortified Liquid BSFFF	19.8±1.5ab	11.4±1.28a	2.3±0.41a	1.88±0.87ab	22.5±5.8ab	10.0±2.31a	2.4±0.62a	3.52±0.5c
Significance level	*	ns	ns	*	*	ns	ns	***
Df	5	5	5	5	5	5	5	5
F/ χ2value	10.6**^+^**	2.0	2.3	12.5**^+^**	13.7**^+^**	2.0	2.3	16.1

*p<0.05, ***p < 0.001, ns, not significant (p ≥ 0.05), **^+^**, chi square value. Control, no fertilizer amendment; Safi, commercial organic fertilizer; BSFFF, black soldier fly frass fertilizer. Per column, mean (±standard error) followed by same letters are not significantly different at p<0.05.

#### 3.2.2 Potassium uptake

There was no significant difference in potassium uptake of broccoli grown using different fertilizer amendments (**Table 2**). During season 2023A, the potassium uptake ranged between 7.8 and 11.8 kg ha^-1^ for the control treatment and chitin-fortified solid BSFFF, respectively. In season 2023B, the lowest potassium uptake (7.8 kg ha^-1^) was recorded in broccoli grown using Safi fertilizer while the highest (11.5 kg ha^-1^) was achieved by broccoli grown using chitin-fortified solid BSFFF.

#### 3.2.3 Phosphorus uptake

The fertilizer amendments did not significantly influence the phosphorus uptake of broccoli (**Table 2**). The phosphorus uptake ranged between 1.4 and 2.8 kg ha^-1^ during season 2023A, and 1.7 – 2.6 kg ha^-1^ during season 2023B.

#### 3.2.4 Agronomic nitrogen use efficiency

The fertilizer amendments showed significant improvements in agronomic nitrogen efficiency in both seasons (**Table 2**). The fertilizer amendments significantly increased agronomic nitrogen efficiency by 1.6 – 3.3-fold and 1.1 – 4.1-fold higher in seasons 2023A and 2023B, respectively, in comparison to the control. The BSFFF amendments significantly increased agronomic nitrogen efficiency by 1.8 – 3.6-fold and 1.5 – 4.6-fold higher compared to Safi in seasons 2023A and 2023B, respectively. Plots amended with chitin-fortified solid BSFFF and solid BSFFF produced broccoli with 3.3-fold and 4.1-fold higher nitrogen uptake in seasons 2023A and 2023B, respectively.

Broccoli grown with chitin-fortified liquid BSFFF had higher agronomic nitrogen efficiency by 7% and 249% than with liquid BSFFF during seasons 2023A and 2023B, respectively. The performance of both solid and chitin-fortified solid BSFFF varied in both seasons when compared to each other. In season 2023A, chitin-fortified solid BSFFF produced broccoli with 111% higher agronomic nitrogen efficiency than the solid BSFFF. However, in season 2023B, solid BSFFF produced broccoli with an agronomic efficiency that was 34% higher than that achieved with chitin-fortified solid BSFFF.

### 3.3 Yield parameters of broccoli grown in soil amended with BSF frass products and commercial organic fertilizer

#### 3.3.1 Number of heads

**Figure 3:**
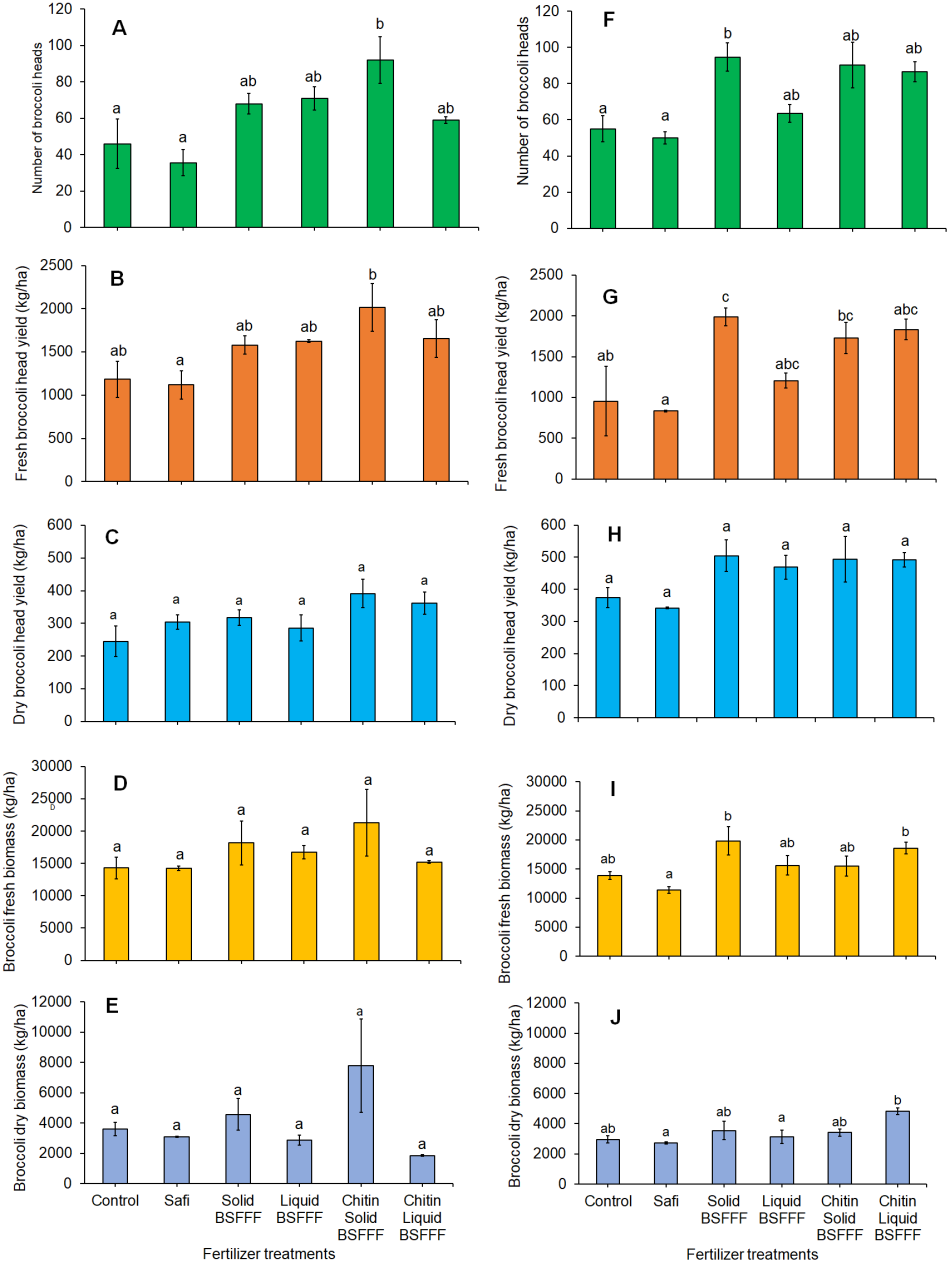
Effects of BSF frass fertilizers and commercial fertilizer on number of broccoli heads **(A, F)**, broccoli fresh yield **(B, G)**, broccoli dry yield **(C, H)**, broccoli fresh biomass **(D, I)**, and broccoli dry biomass **(E, J)** during Season 2023A **(A-E)** and Season 2023B **(F-J)**. Control, no fertilizer amendment; Safi, commercial organic fertilizer; solid BSFFF; liquid BSFFF; chitin-fortified solid BSFFF; chitin-fortified liquid BSFFF. Per panel, bars followed by the same lower case letter(s) are not significantly different at p ≤ 0.05.

The number of broccoli heads varied significantly due to fertilizer treatments during both growing seasons (season 2023A: F = 5.00, df = 5, p< 0.05, season 2023B: F= 5.64, df = 5, p< 0.01). Compared to the control, the different forms of BSFFF increased broccoli head count by 28-100%, and 16 – 72% in seasons 2023A and 2023B, respectively (**Figures 3A, F**). The number of broccoli heads achieved using BSFFF was significantly higher than the value achieved using Safi treatment by 65 – 158% during season 2023A and 27 – 89% in season 2023B.

Chitin-fortified solid BSFFF and non-fortified solid BSFFF produced significantly higher (p< 0.05) broccoli heads compared to all the other treatments during seasons 2023A (by 158%) and 2023B (by 89%). In season 2023A, the application of solid BSFFF produced 49% higher broccoli heads relative to liquid BSFFF in season 2023A. Chitin-fortified solid BSFFF yielded 35% more broccoli heads compared to solid BSFFF during season 2023A. Conversely, liquid BSFFF produced 20% more heads than chitin-fortifies liquid BSFFF in the same season.

#### 3.3.2 Broccoli yield and total biomass

The fertilizer amendments significantly influenced the fresh broccoli head yield (season 2023A: F = 3.2, df = 5, p< 0.05, season 2023B: F = 5.66, df = 5, p< 0.01) but not dry yield (season 2023A: F=2.12, df = 5, P=0.133, season 2023B: F=2.29, df = 5, p = 0.11). The BSFFF treatments significantly increased fresh broccoli head yield by 33 – 70% in season 2023A and 26 – 108% during season 2023B, compared to the control. The same fertilizer treatments also produced higher fresh broccoli head yield than Safi in season 2023A (41 – 80%) and season 2023B (45 – 138%) (**Figures 3B, G**). During season 2023B, the application of solid BSFFF enhanced fresh broccoli head yield by 65% relative to liquid BSFFF, and 15% compared to chitin-fortified solid BSFFF. However, in season 2023A, chitin-fortified solid BSFFF produced 28% higher fresh head yield than solid BSFFF. It was noted that chitin-fortified liquid BSFFF increased fresh head yield by 2% in season 2023A and 54% in season 2023B, compared to liquid BSFFF.

For dry broccoli head yield, values ranged from 244.8 to 391.4 kg ha^-1^ in Season 2023A, with chitin fortified solid BSFFF and the control achieving the highest and lowest values, respectively (**Figures 3C, H**). In season 2023B, the dry yield ranged between 342 and 507 kg ha^-1^ with solid BSFFF achieving the highest value and Safi the lowest.

The fertilizer treatments significantly increased broccoli fresh biomass (season 2023A: χ2 = 5.34, df = 5, p = 0.37, season 2023B: F = 4.2, df = 5, p< 0.05) (**Figures 3D, I**) during season 2023B only, and dry biomass during both seasons (season 2023A: χ2 = 11.88, df = 5, p< 0.05, season 2023B: χ2 = 22.45, df = 5, p< 0.001) (**Figures 3E, J**). The BSFFF formulations significantly increased broccoli fresh by 12 – 43% compared to control, and by 36 – 74% relative to Safi in season 2023B (**Figures 3D, I**). During the same season, the solid BSFFF produced 27% more fresh biomass than the liquid BSFFF while chitin-fortified liquid BSFFF outperformed liquid BSFFF by 19%. However, when comparing the solid formulations, the solid BSFFF yielded 28% more fresh biomass than the chitin-fortified solid BSFFF in season 2023B and solid BSFFF was the most effective of all treatments during season 2023B.

The BSFFF amendments significantly increased the broccoli dry biomass by 27 – 117% and 15 – 77% compared to the control during the seasons 2023A and 2023B, respectively, and by 48-151% and 6-63% as relative to Safi in seasons 2023A and 2023B, respectively (**Figures 3E, J**).

In season 2023A, solid BSFFF enhanced broccoli dry biomass by 59% compared to liquid BSFFF, while in season 2023B, the increase was 13%. Also, the solid BSFFF outperformed chitin-fortified solid BSFFF by 4% in season 2023B. However, the opposite trend was observed in season 2023A, where chitin-fortified solid BSFFF yielded 70% more dry biomass than the non-fortified solid form.

In terms of liquid formulations, liquid BSFFF resulted in 43% higher dry biomass than chitin-fortified liquid BSFFF during season 2023A. In contrast, during season 2023B, chitin-fortified liquid BSFFF outperformed liquid BSFFF by 54%. Among all the treatments, solid BSFFF produced the highest broccoli dry biomass in season 2023A, while chitin-fortified liquid BSFFF achieved the highest biomass in season 2023B.

### 3.4 Nutritional profile of broccoli grown using BSF frass fertilizers and commercial organic fertilizer

#### 3.4.1 Proximate composition

**Figure 4:**
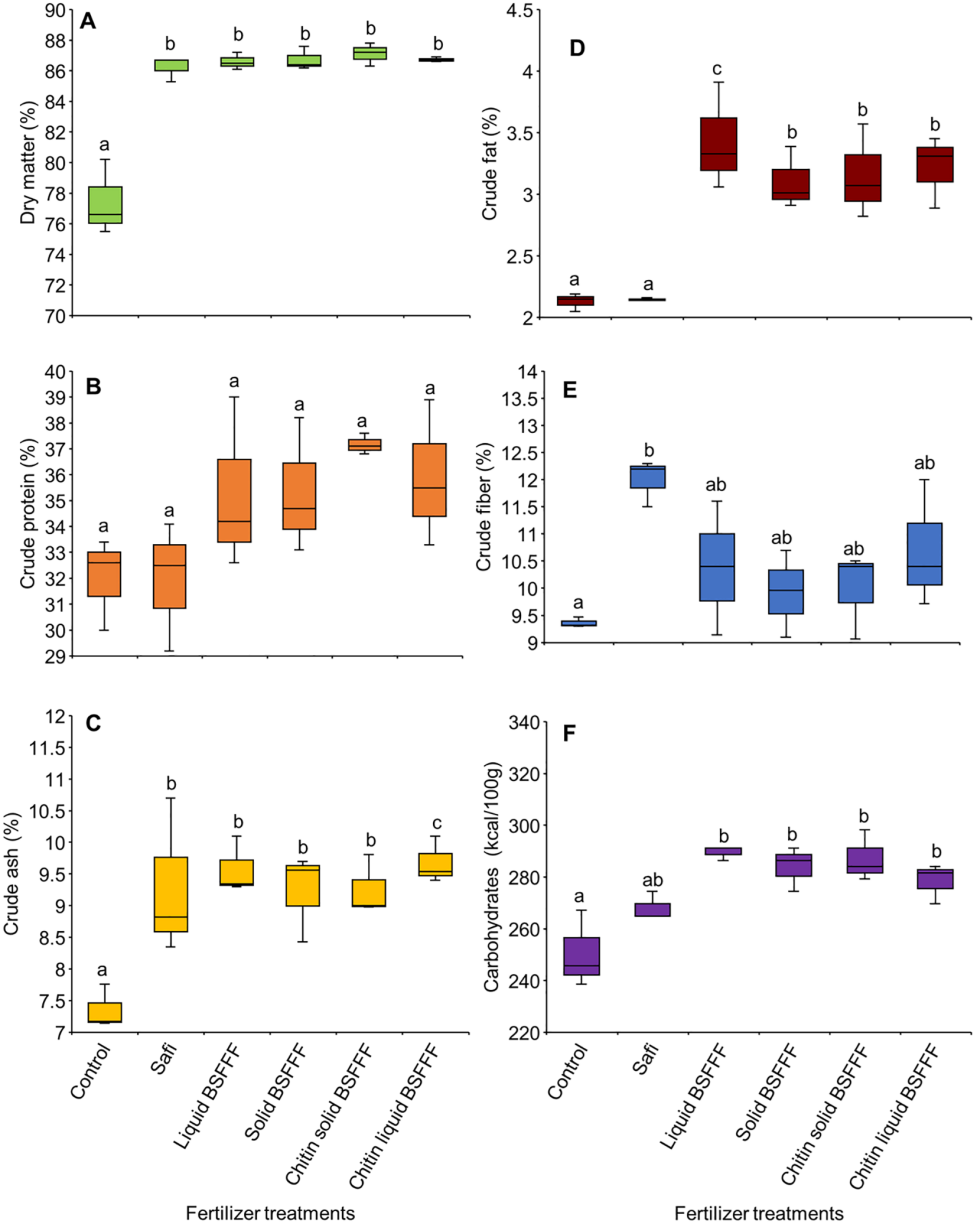
Impact of BSF Frass Fertilizers and commercial fertilizer on broccoli dry matter **(A)**, broccoli crude protein **(B)**, broccoli crude ash **(C)**, broccoli crude fat **(D)**, broccoli crude fiber **(E)**, and broccoli carbohydrate **(F)**. BSFFF, black soldier fly frass fertilizer; Safi, commercial organic fertilizer; control, no fertilizer amendment. Per panel, bars followed by the same lower case letter(s) are not significantly different at p ≤ 0.05.

The fertilizer formulations significantly influenced the dry matter (χ2 = 157.8, df = 5, p< 0.001), ash (F = 4.87, df = 5, p< 0.05), crude fat (F = 12.1, df = 5, p< 0.001), crude fiber (F = 3.39, df = 5, p< 0.05), and carbohydrate (F = 7.96, df = 5, p< 0.01), while crude protein was not significant (F=2.35, df=5, p=0.11) contents of broccoli. The fertilizer amendments significantly increased dry matter by 11 – 12% as compared to the control (**Figure 4A**). Broccoli heads from plots amended with the various BSFFF formulations had 40 – 90% higher dry matter compared to those grown with Safi. The crude protein concentrations ranged from 36.3% to 41.6%, whereby Safi had the lowest value, while chitin-fortified solid BSFFF had the highest value (**Figure 4B**).

Both BSFFF amendments and Safi significantly (p< 0.051) increased the ash concentration by 25-32% relative to the control (**Figure 4C**). The BSFFF amendments improved ash concentration by 3 - 4% when compared with Safi while the liquid BSFFF increased ash content by 4% compared to solid BSFFF. In comparison to control, the fertilizer treatments significantly (p< 0.001) increased fat concentration by 1 – 61% (**Figure 4D**). The different forms of BSFFF amendments also improved fat content by 44 – 60% relative to Safi. Liquid BSFFF enhanced broccoli fat content by 11% compared to solid BSFFF. Additionally, liquid BSFFF produced heads with 7% higher fat content compared to chitin-fortified liquid BSFFF treatment.

The fertilizer amendments significantly (p< 0.001) increased the crude fiber compared with the control by 6 – 28% (**Figure 4E**). Soil amended with Safi produced broccoli heads with the highest crude fiber concentration. Among the BSFFF treatments, liquid BSFF resulted in heads with 5% higher fiber concentration compared to solid BSFFF. Furthermore, both chitin-fortified liquid and chitin-fortified solid BSFFF enhanced fiber levels more effectively than non-fortified counterparts. Similarly, the fertilizer amendments significantly increased the carbohydrate levels of broccoli heads by 7 – 16% and by 4 – 85% compared to the control and Safi, respectively (**Figure 4F**). The carbohydrate concentration among broccoli grown using different BSFFF formulations was comparable, with no major differences between formulations during the study period.

#### 3.4.2 Mineral composition

The application of different BSFFF and commercial fertilizer products caused significant variations in the concentrations of phosphorus, sodium, manganese, boron, zinc, and sulfur but had no significant effect on the levels of potassium, calcium, magnesium, iron, copper, molybdenum, and cobalt (**Table 3**). The potassium content ranged between 2.4% and 2.7%, whereby the control treatment had the lowest value. Fertilizer treatments boosted phosphorus levels by 15 – 19% compared to the control (**Table 3**), with the highest increase recorded in broccoli grown using chitin-fortified liquid BSFFF, and the lowest observed under Safi treatment.

**Table 3:** Mineral composition of broccoli grown in soil amended with BSF frass fertilizers and commercial organic fertilizers.

Treatments	Potassium (%)	Phosphorus (%)	Magnesium (%)	Sodium (ppm)	Calcium (ppm)	Iron (ppm)	Manganese (ppm)	Copper (ppm)	Boron (ppm)	Molybdenum (ppm)	Zinc (ppm)	Sulphur (%)	Cobalt (ppm)
Control	2.37±0.0a	0.52±0.01a	0.23±0.01a	4043±127.01a	0.85±0.1a	216.0±58.1a	57.5±7.1a	3.18±0.12a	22.7±0.96a	6.53±0.42a	56.7±1.11a	1.45±0.02a	0.1±0.001a
Safi	2.66±0.1a	0.60±0.02b	0.23±0.01a	5827±473.7b	1.15±0.03a	641.3±267.3a	95.2±12.0c	3.51±0.03a	27.7±0.96bc	8.04±0.14a	60.37±1.3ab	1.59±0.03d	0.17±0.04a
Solid BSFFF	2.65±0.1a	0.60±0.0b	0.24±0.01a	4725±543.0ab	0.96±0.02a	328.8±13.0a	77.8±2.8abc	3.91±0.07a	31.1±1.07c	7.57±0.14a	62.67±1.3ab	1.44±0.99a	0.15±0.03a
Liquid BSFFF	2.61±0.1a	0.61±0.01b	0.25±0.01a	5750±369.5ab	1.18±0.01a	205.0±11.6a	64.6±0.2ab	4.09±0.19a	29.5±1.21bc	7.66±0.48a	64.07±1.7b	1.56±0.01cd	0.1±0.00a
Chitin-fortified solid BSFFF	2.69±0.1a	0.60±0.01b	0.25±0.01a	5520±184.6ab	1.03±0.1a	246.7±24.0a	67.9±3.1abc	3.86±0.04a	26.6±1.1b	7.79±0.4a	66.37±1.88b	1.49±0.01ab	0.14±0.02a
Chitin-fortified liquid BSFFF	2.61±0.1a	0.62±0.02b	0.25±0.01a	4895±303.1ab	1.09±0.1a	558.0±143.8a	96.4±0.4bc	3.83±0.18a	29.6±1.41bc	7.99±0.24a	61.77±1.47ab	1.51±0.01bc	0.14±0.03a
Significance level	ns	***	ns	*	ns	ns	**	ns	***	ns	***	***	ns
Df	5	5	5	5	5	5	5	5	5	5	5	5	5
F/χ2 value	12.2	35.5**^+^**	0.54	3.7	3.1	11.0**^+^**	5.7	1.9	84.3	10.6	24.4	127.6	4.19

*p<0.05, **p<0.01, ***p<0.001, ns, not significant (p ≥0.05), **^+^** chi square value. Control, no fertilizer amendment; Safi, commercial organic fertilizer; BSFFF, black soldier fly frass fertilizer. Per column, mean (±standard error) followed by same letters are not significantly different at p<0.05.

The magnesium levels ranged between 0.23 and 0.25% in broccoli from control and BSFFF treatments, respectively. Safi significantly increased the sodium levels in broccoli by 44% relative to the control. The calcium and iron levels ranged from 0.85 to 1.18 ppm and 216 to 558 ppm, respectively. The manganese levels were notably higher in broccoli grown in soils soil amended with Safi and chitin-fortified liquid BSFFF, achieving 66 – 68% and 47 – 49% increases, respectively, compared to the values obtained under control and liquid BSFFF treatments.

The BSFFF fertilizer treatments significantly increased the copper levels of broccoli by 20 – 29% relative to the control (**Table 3**), with the highest increases observed under liquid BSFFF. On the other hand, all fertilizer treatments caused significant increases in boron levels by 17 – 37% compared to unamended soil, with broccoli grown in plots amended with solid BSFFF and chitin-fortified solid BSFF achieving 11.3 – 11.7-fold significantly higher boron concentrations than crops grown in unfertilized soil. Additionally, solid BSFFF-amended soils enhanced boron levels of broccoli by 17% compared to chitin-fortified solid BSFFF.

The application of the liquid BSFFF and chitin-fortified solid BSFFF improved the zinc concentration of broccoli by 13% and 17%, respectively, relative to the control. On the other hand, the application of liquid BSFF, chitin-fortified liquid BSFF, and Safi significantly increased the sulfur levels of broccoli by 7.6 – 10.5%, 4.1 – 4.9%, and 9.7 – 10.4%, respectively, compared to control and solid BSFFF. The molybdenum and cobalt levels were 6.1 – 8.0 ppm and 0.1 – 0.17 ppm, with the highest values recorded in broccoli grown in soil amended with Safi and the lowest in unamended soil.

### 3.5 Profitability of broccoli production using BSFFF and commercial organic fertilizer

The net income, gross margin, benefit-to-cost ratio (BCR), and return on investment (ROI) of broccoli production are presented in **Table 4**. Broccoli grown in plots amended with chitin-fortified solid and chitin-fortified liquid BSFFF generated higher net income compared to the vegetables grown in unfertilized plots. The net income yielded by broccoli grown using liquid BSFFF, chitin-fortified solid BSFFF and chitin-fortified liquid BSFFF was 18.9-fold, 25.7-fold, 19.5-fold higher than the values achieved with Safi fertilizer during season 2023A. Furthermore, in the same season, chitin-fortified solid BSFFF (30.5-fold) and chitin-fortified liquid BSFFF (2.9%) yielded significantly higher net income than non-fortified formulations (solid BSFFF and liquid BSFFF). Economically, the chitin-fortified solid BSFFF was the most effective treatment during 2023A which coincided with the long rains, whereas, the liquid formulation was more profitable during 2023B, characterized by short rains.

**Table 4:** Economic value of broccoli production using different BSF frass fertilizer products and commercial organic fertilizer.

Season	Treatment	Net income (USD ha^-1^)	Gross Margin (%)	Benefit cost ratio	Return on investment (%)
Season 2023A	Control	2643.4±282.1bc	97.2±0.3c	36.0±3.8b	3602.9±384.5b
	Safi	143.1±438.0a	1.1±12.7a	0.05±0.2a	5.0±15.4a
	Solid BSFFF	120.3±285.3ab	28.8±4.9a	0.42±0.1a	41.7 ±9.5a
	Liquid BSFFF	2705.2±40.5bc	62.4±0.4b	1.66 ±0.0a	166.0±2.5a
	Chitin-fortified solid BSFFF	3673.0±7.4c	67.0±4.7b	2.15±0.4a	215.2±43.2a
	Chitin-fortified liquid BSFFF	2784.8±582.8bc	61.7±5.2b	1.71±0.4a	170.9±35.8a
	*Significance level*	***	***	***	***
	*Df*	*5*	*5*	*5*	*5*
	*F/ χ2 value*	*7.9*	*28.7*	*402.6* **^+^**	*402.3* **^+^**
Season 2023B	Control	2298.7±981.7c	93.0±5.0c	33.1±0.2b	3307.2±1412.4b
	Safi	-2203.4±35.4b	-88.0±2.7a	-0.47±0.0a	-46.8±0.8a
	Solid BSFFF	-6487.0±336.6a	-109.7±11.9a	-0.52±0.0a	-52.0±2.7a
	Liquid BSFFF	1804.9±277.7c	47.1±3.9b	0.99±0.2a	99.0±15.2a
	Chitin-fortified solid BSFFF	3065.9±566.7c	57.9±4.7b	2.0±0.2a	202.4±20.8a
	Chitin-fortified liquid BSFFF	3694.2 ±379.8c	66.6±2.3bc	1.4±0.3a	143.4±26.5a
	*Significance level*	***	***	***	***
	*Df*	*5*	*5*	*5*	*5*
	*χ2 value*	*287.8*	*1057.3*	*26.4*	*26.4*

***p<0.001, ns, not significant (p ≥ 0.05), **^+^** chi square value. Control, no fertilizer amendment; Safi, commercial organic fertilizer; BSFFF, black soldier fly frass fertilizer. Per column, mean (±standard error) followed by same letters are not significantly different at p<0.05.

The gross margin, BCR, and ROI exhibited similar trends, with BSFFF treatments outperforming Safi. Additionally, chitin-fortification further enhanced the economic returns of broccoli production compared to non-fortified BSFFF products. Notably, the application of Safi or solid BSFFF was less profitable than the production of broccoli with no fertilizer (control treatment).

## 4 Discussion

### 4.1 Effects of different BSF frass fertilizer products and commercial organic fertilizer on broccoli growth and yield

The improved broccoli growth and its association with different black soldier fly frass fertilizers as compared to commercial organic fertilizer and unfertilized soil has been previously reported using vegetables and other crops (Chavez et al., 2024; Chepkorir et al., 2024; Rejeki et al., 2023; Abiya et al., 2022; Dzepe et al., 2022; Anyega et al., 2021; Ouda and Mahadeen, 2008), and this can be ascribed to the high nutrient levels in black soldier fly frass fertilizer and chitin-rich exuviae that is readily available to plants (Anedo et al., 2025; Beesigamukama et al., 2020a). Furthermore, the BSF frass fertilizer does not have phytotoxicity challenges (Beesigamukama et al., 2021b), and has a low C/N ratio, a high mineralization rate (Adin Yéton et al., 2019), and better synchrony of nutrients for plant growth (Beesigamukama et al., 2020b).

Past studies have also demonstrated the benefits of frass fertilizer in addressing soil acidity and salinity, suppressing pests and pathogens, and enhancing soil microbiota, thereby improving soil health for better plant growth (Kisaakye et al., 2024; Anedo et al., 2025; Beesigamukama et al., 2021b; Poveda, 2021; Poveda et al., 2019; Kagata and Ohgushi, 2012). The chitin fertilizer contained in the BSF frass also enhances soil fertility and microbiota. In leafy plants, dry weight is frequently a good sign of above-net primary productivity (Smart et al., 2017), partly due to increased photosynthetic activity (Mendoza-Tafolla et al., 2019) indicated by high chlorophyll content, and root development (Boudabbous et al., 2023) which are consistent with the findings of this study. Past studies (Kisaakye et al., 2024) have reported improved root development in vegetables grown with chitin-fortified black soldier fly frass fertilizer. The enhanced plant growth associated with chitin-fortified fertilizers and BSFFF could be attributed to the synthesis of amino acids that play an active role in plant growth and tolerance to environmental stresses (Trovato et al., 2021).

The higher broccoli growth and yield achieved in the second season can be attributed to residual benefits of the BSFFF amendments and higher rainfall, which enhanced the higher nutrient release and uptake as reported in the study. This also caused the leaves in season 2023B to mature faster than those in season 2023A, thus the difference in peaks of the graphs. The high nitrogen, phosphorus and potassium uptake of broccoli grown in soil amended with BSF frass fertilizer, compared to Safi has been previously reported (Chepkorir et al., 2024; Anyega et al., 2021; Beesigamukama et al., 2020a) and can be attributed to better root formulation linked to phosphorus availability within the rhizosphere (Beesigamukama et al., 2021b).

Soils amended with BSF frass fertilizer improves mineral nitrogen in the root zone, resulting in higher nitrogen uptake and crop yields (Beesigamukama et al., 2021b). Therefore, the higher agronomic nitrogen efficiency achieved using frass fertilizer products when compared to Safi, accrues from the multiple benefits of these novel fertilizer products on soil health and aligns with previous studies (Anyega et al., 2021; Beesigamukama et al., 2020a). Our findings show that farmers would need to apply fewer quantities to realize higher increases in crop yields (Beesigamukama et al., 2022b), hence reducing the burden of high fertilizer purchase costs. It should be noted that high prices are one of the hindrances to fertilizer use in Africa and most developing countries. Therefore, adopting high-quality frass fertilizer could provide a sustainable solution to this challenge and transform agri-food systems.

The differences in broccoli growth and yield observed due to the different BSFFF products highlight the crucial role of fertilizer source and quality in influencing soil health, crop productivity, and overall food security. The higher broccoli yield achieved using BSF frass fertilizers may be attributed to their the additional benefits, such as pest and disease suppression, which is not typically provided by conventional fertilizers like Safi (Anedo et al., 2025; Tanga and Kababu, 2023; Wantulla et al., 2023; Barragán-Fonseca et al., 2022). It should be noted that due to climate change and soil degradation, crop production faces several abiotic challenges and adoption of multipurpose inputs such as insect frass fertilizers provide a holistic solution. These inputs not only supply essential nutrients but also contribute to pests and disease control, improve soil moisture retention, and enhance biodiversity (Tanga and Kababu, 2023). The regenerative insect frass fertilizers also contribute to safe food production due to reduced pesticide and agrochemical residues in food crops.

The differences in the performance of solid and liquid frass fertilizers observed can be attributed to the distinct characteristics of each product. The liquid frass fertilizers contain readily available soluble nutrients for plant uptake compared to the solid which requires time, water, and microbes for the mineralization process. The attribute of fast nutrient release associated with liquid frass fertilizers enables them to rapidly address nutrient deficiencies, even during critical crop growth stages, leading to better growth and yield. Therefore, the superior performance of liquid BSFFF observed during the dry season 2023A could be largely due to its dual-purpose role of supplying both moisture and nutrients, making it valuable during periods of moisture stress. This characteristic positions liquid BSFFF as a promising input for climate-resilient agriculture, helping cropping systems adapt to the impacts of climate change (Abiya et al., 2022; Barragán-Fonseca et al., 2022).

On the other hand, the higher agronomic efficacy of chitin-fortified BSF frass fertilizers compared to non-fortified BSF frass fertilizers can be attributed to the benefits of chitin in enhancing crop growth, nutrient availability, and plant defense through suppression of pests and diseases (Anedo et al., 2025; Kisaakye et al., 2025; Quilliam et al., 2020). Past studies have reported reduced aphid infestation of pests in vegetables grown in soil amended with chitin-fortified fertilizer (Kisaakye et al., 2024). Additionally, chitin-fortified fertilizers also promotes the growth of chitin-degrading microbes, which enhances microbial activity and diversity, which in turn improves soil fertility and soil microbiota (Barragán-Fonseca et al., 2022). Future research should explore the effect of the different forms of black soldier fly frass fertilizers on the management of broccoli pests and diseases to generate accurate recommendations.

### 4.2 Nutritional quality and profitability of broccoli production using different organic fertilizers

The increased concentrations of crude protein, fat, ash, and carbohydrate observed in this study following the application of the different forms of BSFFF shows that, alongside vegetable yield, BSFFF is efficient in boosting the nutritional quality of vegetable crops. These results are align with previous studies that reported improved nutritional profiles of different food crops grown in soils amended with insect frass fertilizers (Anyega et al., 2021; Yoder and Davis, 2020; Meena et al., 2018; Mukta et al., 2016; Chakwizira et al., 2015). The observed improved nutritional quality of broccoli could be associated with nutrient uptake, as discussed in section 3.1. In particular, the enhanced levels of minerals such as potassium, phosphorus, calcium, and manganese corroborate earlier reports on use of BSF frass fertilizer (Menino et al., 2021) and highlight the role of BSF frass fertilizer in eliminating hidden hunger and malnutrition, contributing to sustainable development goal to zero hunger. Moreover, recent studies have revealed a high willingness to buy vegetable foods produced using insect frass fertilizer and animal feed on insect-based feeds (Traore et al., 2024), paving the way for full-scale development of insect-based value chains. However, to ensure consumer acceptance and marketability, organoleptic tests are necessary to evaluate taste, texture, and overall appeal, thereby supporting the refinement of insect-based fertilizers and the formulation of recommendations for producing nutritious and palatable food.

The higher economic returns observed with the application of different forms of BSFFF formulations, compared with Safi could be largely attributed to their high quality and affordability of BSFFF, as previously reported (Chepkorir et al., 2024; Tanga et al., 2021). In particular, the higher net income, gross margin, benefit-to-cost, and return on investment achieved with chitin-fortified frass fertilizers relative to other fertilizer products assessed could be largely attributed to dual benefits of controlling pests and diseases while supplying nutrients (Anedo et al., 2025; Kisaakye et al., 2024). Furthermore, the enhanced profitability of liquid BSFFF formulations during the dry season suggests additional advantages, such as contributing moisture during periods of water stress. This not only mitigates irrigation costs but also improves crop performance and revenues. The liquid formulations are also more portable than their solid counterparts, reducing transportation costs and making them more accessible to smallholder farmers (Tanga and Kababu, 2023). These findings show that by adopting diversified BSF frass fertilizers, farmers can reduce fertilizer purchase costs, increase income, and contribute to improved soil health, and ultimately improved food security (Beesigamukama et al., 2022b).

## 5 Conclusion

Our study has demonstrated the high efficacy of the different BSFFF products in boosting vegetable productivity better than the conventional organic fertilizer assessed. For higher broccoli yield, nutritional quality, and profitability, chitin-fortified BSF frass fertilizers are recommended. Our findings show that the liquid and chitin-fortified BSF frass fertilizer products can provide sustainable substitutes for most commercial fertilizers, thus reducing the burden of costly fertilizers and ensuring planetary health. The liquid BSF frass fertilizer should be applied during the dry season to improve crop resilience to moisture stress and minimize nutrient leaching during the rainy season. The adoption of various forms of BSF frass fertilizers presents a more sustainable approach to agriculture, accelerating the transition to circular and regenerative farming practices. Future studies will be necessary to validate the yield benefits on other crops and evaluate the effects of different BSF frass fertilizer products on soil health and management of broccoli pests and diseases.

## Data Availability

The original contributions presented in the study are included in the article/Supplementary Material. Further inquiries can be directed to the corresponding authors.
